# Comparison of Baseline versus Posttreatment Left Ventricular Ejection Fraction in Patients with Acute Decompensated Heart Failure for Predicting Cardiovascular Outcome: Implications from Single-Center Systolic Heart Failure Cohort

**DOI:** 10.1371/journal.pone.0145514

**Published:** 2016-01-11

**Authors:** Jih-Kai Yeh, Yuan-Chuan Hsiao, Cian-Ruei Jian, Chao-Hung Wang, Ming-Shien Wen, Chi-Tai Kuo, Feng-Chun Tsai, Victor Chien-Chia Wu, Tien-Hsing Chen

**Affiliations:** 1 Department of Cardiology, Chang Gung Memorial Hospital, Linkou Medical Center, Taoyuan City, Taiwan; 2 Chang Gung University College of Medicine, Taoyuan, Taiwan; 3 Heart Failure Center, Division of Cardiology, Department of Internal Medicine, Chang Gung Memorial Hospital, Keelung, Taiwan; 4 Department of Cardiothoracic and Vascular Surgery, Chang Gung Memorial Hospital, Linkou Medical Center, Taoyuan City, Taiwan; 5 Department of Cardiology, Chang Gung Hospital, Xiamen, China; University of Miami School of Medicine, UNITED STATES

## Abstract

**Aims:**

The prognostic values of left ventricular ejection fraction (LVEF) during heart failure (HF) with acute decompensation or after optimal treatment have not been extensively studied. We hypothesized that posttreatment LVEF has superior predictive value for long-term prognosis than LVEF at admission does.

**Methods and Results:**

In Protocol 1, 428 acute decompensated HF (ADHF) patients with LVEF ≤35% in a tertiary medical center were enrolled and followed for a mean period of 34.7 ± 10.8 months. The primary and secondary end points were all-cause mortality and HF readmission, respectively. In total, 86 deaths and 240 HF readmissions were recorded. The predictive values of baseline LVEF at admission and LVEF 6 months posttreatment were analyzed and compared. The posttreatment LVEFs were predictive for future events (*P* = 0.01 for all-cause mortality, *P* < 0.001 for HF readmission), but the baseline LVEFs were not. In Protocol 2, the outcomes of patients with improved LVEF (change of LVEF: ≥+10%), unchanged LVEF (change of LVEF: –10% to +10%), and reduced LVEF (change of LVEF: ≤–10%) were analyzed and compared. Improved LVEF occurred in 171 patients and was associated with a superior long-term prognosis among all groups (*P* = 0.02 for all-cause mortality, *P* < 0.001 for HF readmission). In Protocol 3, independent predictors of improved LVEF were analyzed, and baseline LV end-diastolic dimension (LVEDD) was identified as a powerful predictor in ADHF patients (*P* < 0.001).

**Conclusions:**

In patients with ADHF, posttreatment LVEF but not baseline LVEF had prognostic power. Improved LVEF was associated with superior long-term prognosis, and baseline LVEDD identified patients who were more likely to have improved LVEF. Therefore, baseline LVEF should not be considered a relevant prognosis factor in clinical practice for patients with ADHF.

## Introduction

Heart failure (HF) has emerged as a major public health concern. In the United States, the prevalence of HF is 5.7 million people, and approximately 870,000 new cases are reported annually [1]. Although clinical trials have established numerous therapies for improving the clinical outcomes of patients with HF and reduced left ventricular (LV) ejection fraction (LVEF), the overall prognosis remains poor, with mortality exceeding 50% at 5 years with a high rate of rehospitalization (up to 50% in 1 y), placing a financial burden on national health care systems [2–5]. Using echocardiography to measure LVEF is noninvasive, and this technique is commonly performed to assess myocardial function for guiding clinical therapeutic strategy in patients with HF [6].

In patients with chronic systolic HF, LV function assessed using LVEF is a crucial determinant of cardiovascular outcomes [7,8]. However, the precise association of LVEF with cardiovascular outcomes in patients with acute decompensated HF is controversial [9]. Because the LVEF measure is load-dependent and varies with hemodynamic status, it may underestimate or overestimate true myocardial function in various pathophysiologic conditions and precipitants of acute decompensation. A prospective study reported that LVEF was weakly correlated with hemodynamic measures and clinical outcomes in patients with acute HF [10]. In practice, myocardial recovery is possible, and thus significant LVEF changes occur after evidence-based HF therapy. Previous studies have not determined the exact relationship between baseline and posttreatment LVEF and cardiovascular outcomes, and the predictors of LV systolic function improvement remain elusive [11–17].

Therefore, this study evaluated the following: (1) the prognostic value of baseline LVEF in patients with ADHF compared with that of patients with LVEF 6 months posttreatment, (2) the prognostic value of the change in LVEF obtained from the difference between baseline and posttreatment LVEF, and (3) the predictors of patients with improved LVEF.

## Methods

### Study Population and End Points

The study conformed to the principles of the Declaration of Helsinki and was approved by Chang Gung Memorial Hospital Institutional Review Board. As written informed consent was not necessary and therefore not obtained for review of medical records, the patient records and information were anonymized and deidentified prior to analysis

In Protocol 1, 811 consecutive patients admitted to our hospital from January to December 2010 with a principal diagnosis of ADHF and baseline echocardiographic LVEF ≤35% were enrolled. For each admission, the presence of ADHF was confirmed by 2 authors (Yuan-Chuan Hsiao and Cian-Ruei Jian), who followed the Framingham criteria, reviewed all patient charts independently, and reached a consensus on each case. All patients were treated with angiotensin-converting enzyme inhibitors (ACEIs) or angiotensin-receptor blockers (ARBs), aldosterone antagonists, and β blockers if there was no contraindication, and these medications were continued and up-titrated during follow-up visits. For patients with de novo HF, detailed clinical information acquirement, laboratory studies, electrocardiography (ECG), echocardiography, nuclear myocardial perfusion scanning, cardiac magnetic resonance imaging, and/or coronary angiography were implemented to maximize HF etiology precision. In addition, causes and precipitants for ADHF were identified, and treatment with corresponsive therapies according to the contemporary HF guidelines [6] was applied, including coronary revascularization for ischemic heart disease (IHD), valvular surgery for severe symptomatic valvular heart disease, radiofrequency ablation for tachyarrhythmia, and device implantation for bradycardia therapy, cardiac resynchronized therapy, and/or sudden death prevention. The primary and secondary end points were defined as all-cause mortality and HF readmission. Medical records were reviewed for each patient after enrollment and were searched to identify deaths and subsequent admission with a principal diagnosis of congestive HF; these outcomes were confirmed during regular visits and telephone contact with the patients or their relatives through Dec. 31, 2013. Patients with survival <6 months, loss to follow-up, or unavailable posttreatment LVEF data were excluded. The prognostic values of baseline and posttreatment LVEF for the study end points were compared. In Protocol 2, according to the change in LVEF, the patients were separated into 3 groups: improved LVEF (change of LVEF: ≥+10%), unchanged LVEF (change of LVEF: –10% to +10%), and reduced LVEF (change of LVEF: ≤–10%). The prognostic value of improved LVEF end points was assessed. In Protocol 3, independent variables that were associated with improved LVEF were evaluated.

### Data Collection and Variables

Data recorded on the abstraction form included clinical symptoms, New York Heart Association functional class (NYHA Fc), comorbidities, laboratory data, admission blood pressure and heart rate, discharge medications, and 12-lead ECG results. The recorded comorbidities were hypertension (HT), diabetes mellitus (DM), chronic obstructive pulmonary disease (COPD), stroke, cirrhosis, and end-stage renal disease (ESRD). Specifically, ESRD was defined as estimated GFR <15 mL/min/1.73m^2^ with long-term dialysis therapy. IHD was defined as history of myocardial infarction or angina with documented myocardial ischemia identified using stress tests, a pathologic Q wave on an ECG, or significant >50% stenosis in one or more coronary arteries on a coronary angiogram. Baseline echocardiography evaluations were conducted at admission, and LVEF was measured using the M-mode or modified Simpson’s method, as recommended by the American Society of Echocardiography [18]. Posttreatment LVEF was defined as echocardiographic assessment of at least 6 months after enrollment.

### Statistical Analysis

The chi-square test or Fisher’s exact test was used to evaluate dichotomous variables, and the Student *t* test was used for continuous variables. We analyzed the association of candidate variables with all-cause mortality and HF readmission in a univariate Cox proportional hazards regression model and assessed the predictive value of baseline LVEF, posttreatment LVEF, and improved LVEF >10% after adjusting for baseline clinical characteristics. The patients were divided into 3 groups according to baseline or posttreatment LVEF tertiles. Kaplan–Meier curves with log-rank tests were used to construct cumulative survival of all-cause mortality and HF readmission rates for estimating the differences among the 3 groups. Variables that differed significantly among the improved, unchanged, or reduced LVEF groups were analyzed using a multivariate logistic regression model to identify the independent predictors of improved LVEF. The differences in all-cause mortality and HF readmission among the improved, unchanged, and reduced LVEF groups were also depicted using Kaplan–Meier curves with log-rank tests. A *P* value below .05 indicated statistical significance. Two-tailed analysis was applicable to all variable assessments. Statistical analysis was performed using SPSS 18.0 (SPSS Inc., Chicago, IL). Incremental values of baseline LVEF, posttreatment LVEF, and LVEF changes were assessed in 3 modeling steps by using nested regression models. The first step consisted of fitting a multivariate base model of age, sex, HT, DM, IHD, COPD, ESRD, and NYHA Fc. Either baseline LVEF, posttreatment LVEF, or LVEF change was included in the second step. The change in overall log likelihood ratio χ^2^ was used to assess the increase in predictive power after adding LVEF.

## Results

### Protocol 1: Outcomes and Predictors of Baseline LVEF and Posttreatment LVEF

**Fig 1:**
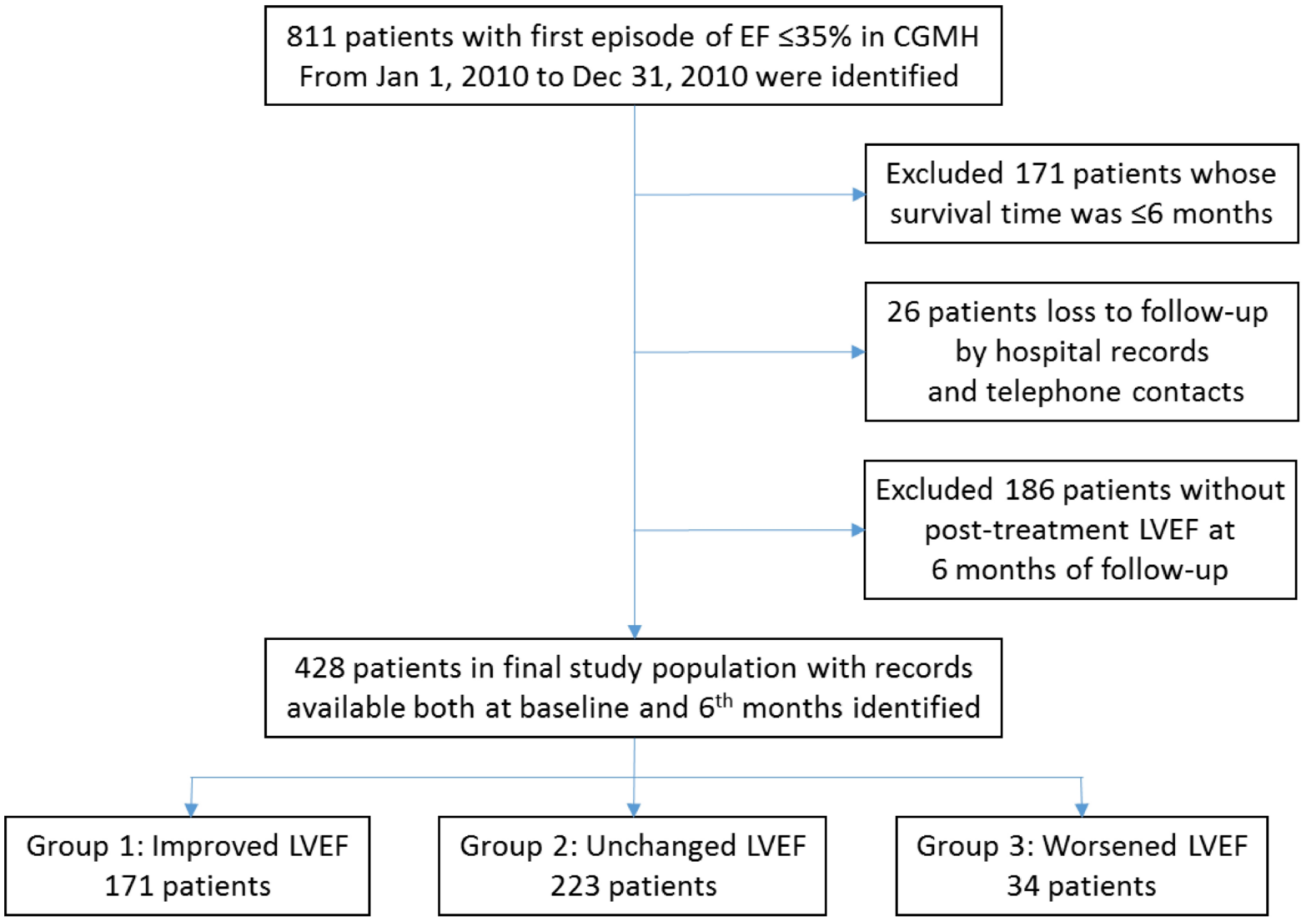
Flow chart of patient enrollment and study eligibility.

Of the 811 patients with ADHF that we initially enrolled, we excluded 171 who survived for less than 6 months after enrollment, 26 patients who were lost to follow-up, and 186 patients for whom 6-month posttreatment echocardiography data were unavailable. The final study population consisted of 428 patients (mean age: 64 ± 15 y), 304 of whom were male (Fig 1). Baseline characteristics are provided in Table 1. In the follow-up period (mean: 34.7 ± 10 mo), 86 cases of all-cause mortality (20.1%) and 240 HF readmissions (56.1%) were recorded. Tables 2 and 3 present the baseline characteristics of the all-cause mortality and HF readmission groups, respectively. The patients who died tended to be older, to have been treated with diuretics, and to have more severe HF symptoms (NYHA function class III/IV), COPD, conduction disturbance QRS duration >120 ms, lower posttreatment LVEF, and higher serum creatinine levels.

**Table 1:** Baseline characteristics of study population (*n* = 428).

Characteristics	All patient*
Gender (male)	304 (71.0%)
Age (year)	64±15
NYHA Fc III/IV	230 (53.7%)
Comorbidity	
IHD	181 (42.3%)
HT	224 (52.3%)
DM	132 (30.8%)
Stroke	49 (11.4%)
COPD	55 (12.9%)
ESRD	22 (5.1%)
Liver cirrhosis	15 (3.5%)
Laboratory data	
Hemoglobin (g/dL)	12.7±2.3
BUN (mg/dL)	28.1±18.4
Creatinine (mg/dL)	1.67±1.87
Albumin (g/dL)	3.7±0.5
Sodium (mEq/L)	139.2±3.7
Uric acid (mg/dL)	7.7±2.4
Admission vital signs	
SBP (mmHg)	126±22
DBP (mmHg)	74±15
Heart rate (bpm)	86±20
Echo parameters	
LA diameter (mm)	45.1±8.5
LVEDD (mm)	61.2±8.7
Baseline LVEF %	26.9±6.1
Post-treatment LVEF%	35.1±14.1
ECG characteristics	
Atrial fibrillation	58 (13.6%)
QRS duration >120 ms	138 (32.2%)
Complete LBBB	41 (9.6%)
Medications	
Warfarin	50 (11.7%)
Aspirin	182 (42.5%)
Clopidogrel	66 (15.4%)
ACEI or ARB	319 (74.5%)
Beta blocker	298 (69.6%)
Digoxin	105 (24.5%)
Diuretics	314 (73.4%)
Device therapy	
PPM	22 (5.1%)
ICD	14 (3.3%)
CRT	28 (6.5%)
CRT-D	33 (7.7%)

*Categorical variables are expressed in number and percentage; continuous variables are presented as mean ± standard deviation.

ACEI, angiotensin converting enzyme inhibitor; AF, atrial fibrillation; ARB, angiotensin II receptor blocker; CCB, calcium channel blocker; COPD, chronic obstructive pulmonary disease; CRT, cardiac resynchronization therapy; CRT-D, cardiac resynchronization therapy-defibrillator; DBP, diastolic blood pressure; DM, diabetes mellitus; ESRD, end-stage renal disease; HT, hypertension; ICD, implantable cardioverter defibrillator; IHD, ischemic heart disease; LBBB, left bundle-branch block; LA, left atrial; LV, left ventricular; LVEDD, left ventricular end-diastolic dimension; LVEF, left ventricular ejection fraction; NYHA Fc, New York Heart Association functional class; PPM, permanent pacemaker; SBP, systolic blood pressure.

**Table 2:** Baseline characteristics stratified by all-cause mortality.

Characteristics	Mortality (*n* = 86)	Survival (*n* = 342)	*P*
Gender (male)	62 (72.1)	242 (70.8)	0.894
Age (year)	70±14	62±15	0.000
NYHA Fc III/IV	56 (65.1)	174 (50.9)	0.021
Comorbidity			
HT	51 (59.3)	173 (50.6)	0.184
DM	32 (37.2)	100 (29.2)	0.154
IHD	37 (43.0)	144 (42.1)	0.903
Stroke	14 (16.3)	35 (10.2)	0.130
COPD	18 (20.9)	37 (10.8)	0.018
ESRD	8 (9.3)	14 (4.1)	0.059
Liver cirrhosis	6 (7.0)	9 (2.6)	0.091
Biochemistry			
Cr (mg/dL)	2.16±2.0	1.51±1.7	0.012
Sodium (mEq/L)	139±3.7	139±3.8	0.502
Admission vital signs			
SBP (mmHg)	124±24	127±21	0.311
DBP (mmHg)	71±16	74±15	0.112
Heart rate (bpm)	86±21	86±19	0.909
Echo findings			
LA diameter (mm)	45.6±8.4	44.9±8.6	0.546
LVEDD (mm)	61.7±8.9	61.1±8.7	0.534
Baseline LVEF (%)	26.6±6.0	26.9±6.9	0.226
Post treatment LVEF (%)	30.3±10.7	36.3±14.6	0.000
ECG characteristics			
AF	12 (14.0)	46 (13.5)	0.862
QRS >120ms	34 (42.0)	104 (33.1)	0.151
Medication			
Aspirin	31 (36.0)	151 (44.2)	0.182
Clopidogrel	16 (18.6)	50 (14.6)	0.403
ACEI or ARB	64 (74.4)	255 (74.6)	1.000
Beta blocker	58 (67.4)	240 (70.2)	0.694
Digoxin	16 (18.6)	89 (26.0)	0.164
Diuretics	73 (84.9)	241 (70.5)	0.006
Amiodarone	8 (9.3)	40 (11.7)	0.702
Aldosterone antagonist	21 (24.4)	94 (27.5)	0.683
CCB	6 (7.0)	39 (11.4)	0.325
Statin	29 (33.7)	155 (45.3)	0.067

**Table 3:** Baseline characteristics stratified by HF readmission.

Characteristics	No (*n* = 188)	Readmission (*n* = 240)	*P*
Gender (male)	143 (76.1)	161 (67.1)	0.053
Age (year)	61±16	66±14	0.001
NYHA class III/IV	88 (46.8)	142 (59.2)	0.011
Comorbidity			
HT	76 (40.4)	148 (61.7)	0.000
DM	46 (24.5)	86 (35.8)	0.012
IHD	84 (44.7)	97 (40.4)	0.377
Stroke	23 (12.2)	26 (10.8)	0.650
COPD	13 (6.9)	47 (17.5)	0.001
ESRD	5 (2.7)	17 (7.1)	0.047
Liver cirrhosis	7 (3.7)	8 (3.3)	0.091
Biochemistry			
Cr (mg/dL)	1.41±1.4	1.87±2.0	0.008
Sodium (mEq/L)	139±3.6	139±3.8	0.970
Admission vital signs			
SBP (mmHg)	126±23	126±21	0.858
DBP (mmHg)	74±15	74±15	0.943
Heart rate (bpm)	85±20	87±20	0.342
Echo findings			
LA diameter (mm)	43.6±9.0	46.2±8.0	0.001
LVEDD (mm)	60.9±8.7	61.4±8.8	0.476
Baseline LVEF (%)	27.3±5.7	26.5±6.5	0.196
Post treatment LVEF (%)	38.0±14.6	32.8±13.4	0.000
ECG characteristics			
AF	22 (11.7)	36 (15.0)	0.393
QRS >120ms	51 (30.9)	87 (37.8)	0.165
Medication			
Aspirin	80 (42.6)	102 (42.5)	1.000
Clopidogrel	24 (12.8)	42 (17.5)	0.225
ACEI or ARB	139 (73.9)	180 (75.0)	0.824
Beta blocker	129 (68.6)	169 (70.4)	0.751
Digoxin	46 (24.5)	59 (24.6)	1.000
Diuretics	129 (68.6)	185 (77.1)	0.061
Amiodarone	21 (11.2)	27 (11.3)	1.000
Aldosterone antagonist	46 (24.5)	69 (28.8)	0.380
CCB	18 (9.6)	27 (11.3)	0.636
Statin	76 (40.4)	108 (45.0)	0.376

ACEI, angiotensin converting enzyme inhibitor; AF, atrial fibrillation; ARB, angiotensin II receptor blocker; CCB, calcium channel blocker; COPD, chronic obstructive pulmonary disease; DBP, diastolic blood pressure; DM, diabetes mellitus; ESRD, end-stage renal disease; HT, hypertension; IHD, ischemic heart disease; LBBB, left bundle-branch block; LA, left atrial; LV, left ventricular; LVEDD, left ventricular end-diastolic dimension; LVEF, left ventricular ejection fraction; NYHA Fc, New York Heart Association functional class; SBP, systolic blood pressure.

**Fig 2:**
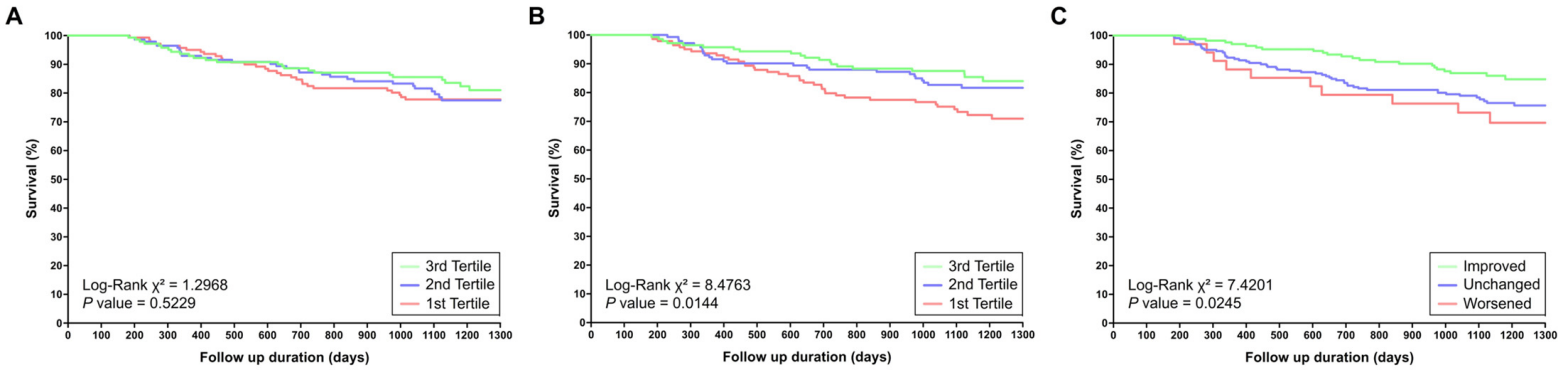
Cumulative percent survival free of all-cause mortality according to baseline LVEF (A), posttreatment LVEF (B), and LVEF change (C). The Kaplan–Meier survival analyses for the patients with HF divided into tertiles by posttreatment LVEF or LVEF change reveal significant differences between the groups in all-cause mortality (*P* = .01). However, all-cause mortality does not differ significantly between the groups according to the baseline LVEF tertiles.

**Fig 3:**
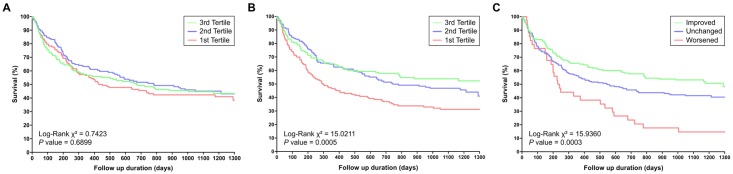
Cumulative percent survival free of HF readmission according to baseline LVEF (A), posttreatment LVEF (B), and LVEF change (C). The Kaplan–Meier survival analyses for the patients with HF divided into tertiles by posttreatment LVEF or LVEF change reveal significant differences between the groups in HF readmission (*P* < .001). However, HF readmission does not differ significantly between the groups according to the baseline LVEF tertiles.

We divided the patients with HF by baseline LVEF and posttreatment LVEF into tertiles and compared them using Kaplan–Meier analysis. The difference between the posttreatment LVEF tertiles for future events was statistically significant (*P* = .01 for all-cause mortality, *P* < .001 for HF readmission), but that between the baseline LVEF tertiles was not (*P* = .52 for all-cause mortality, *P* = .69 for HF readmission) (Figs 2A, 2B and 3A, 3B). In the univariate Cox proportional hazards model, 4 parameters were predictive of all-cause mortality: age, COPD, NYHA Fc III/IV, and posttreatment LVEF. The risk of all-cause mortality increased significantly with age (hazard ratio [HR] = 1.04, *P* < .001), COPD (HR = 1.89, *P* = .028), NYHA Fc III/IV (HR = 1.93, *P* = .004), posttreatment LVEF (HR = 0.68, *P* = .005) (Table 4). In the univariate Cox proportional hazards model, 8 parameters were predictive of HF readmission: age, male sex, HT, DM, COPD, ESRD, NYHA Fc III/IV, and posttreatment EF. However, regardless of all-cause mortality or HF readmission, baseline LVEF did not have a predictive value. In the multivariate Cox proportional hazards model, after adjustment for age, sex, HF, DM, IHD, COPD, ESRD, and NYHA Fc III/IV, posttreatment LVEF remained a statistically significant predictor of future events (HR = 0.39, *P* < .001 for all-cause mortality; HR 0.67, *P* < .001 for HF readmission), but not baseline LVEF (*P* = .17 for all-cause mortality, *P* = .99 for HF readmission) (Table 4; Models 1 and 2).

**Table 4:** Predictors of all-cause mortality and heart failure readmission.

All-Cause Mortality	Univariate Analysis		Multivariate Analysis					
			Model 1		Model 2		Model 3	
	HR (95% CI)	*P*	HR (95% CI)	*P*	HR (95% CI)	*P*	HR (95% CI)	*P*
Age	1.04 (1.03–1.06)	<0.0001*	1.05 (1.03–1.07)	<0.0001*	1.05 (1.03–1.07)	<0.0001*	1.04 (1.03–1.07)	<0.0001*
Sex	1.07 (0.67–1.77)	0.7775	1.26 (0.78–2.11)	0.3430	1.20 (0.74–2.01)	0.4590	1.27 (0.79–2.12)	0.3293
HT	1.34 (0.87–2.08)	0.1858	1.02 (0.65–1.62)	0.9326	1.06 (0.68–1.68)	0.8013	1.08 (0.69–1.71)	0.7333
DM	1.41 (0.90–2.17)	0.1345	1.45 (0.89–2.32)	0.1303	1.45 (0.89–2.33)	0.1325	1.44 (0.89–2.29)	0.1395
IHD	1.01 (0.65–1.55)	0.9649	0.71 (0.45–1.12)	0.1410	0.74 (0.47–1.15)	0.1746	0.75 (0.48–1.17)	0.2121
COPD	1.89 (1.07–3.14)	0.0284*	1.22 (0.67–2.09)	0.5032	1.28 (0.71–2.17)	0.3984	1.39 (0.77–2.36)	0.2603
ESRD	2.20 (0.97–4.28)	0.0568	3.38 (1.45–6.92)	0.0066*	3.31 (1.42–6.80)	0.0076*	3.12 (1.34–6.40)	0.0107*
NHYA Fc	1.93 (1.23–3.07)	0.0036*	1.61 (1.02–2.59)	0.0402*	1.80 (1.14–2.90)	0.0110	1.83 (1.16–2.96)	0.0090*
Baseline LVEF	0.86 (0.66–1.12)	0.2672	0.83 (0.63–1.09)	0.1740				
Post-Treatment LVEF	0.68 (0.52–0.89)	0.0051*			0.39 (0.22–0.67)	0.0008*		
Change of LVEF	0.63 (0.45–0.88)	0.0077*					0.59 (0.42–0.83)	0.0026*
HF Re-Admission	Univariate Analysis		Multivariate Analysis					
			Model 1		Model 2		Model 3	
	HR (95% CI)	*P*	HR (95% CI)	*P*	HR (95% CI)	*P*	HR (95% CI)	*P*
Age	1.02 (1.00–1.03)	<0.0001*	1.01 (1.01–1.02)	0.0022*	1.02 (1.01–1.03)	0.0012*	1.01 (1.00–1.02)	0.0030*
Sex	0.72 (0.55–0.95)	0.0191	0.83 (0.62–1.10)	0.1849	0.75 (0.57–1.00)	0.0489*	0.78 (0.59–1.04)	0.0884
HT	1.77 (1.36–2.30)	<0.0001*	1.62 (1.23–2.14)	0.0005*	1.67 (1.28–2.21)	0.0002*	1.72 (1.31–2.26)	<0.0001*
DM	1.47 (1.12–1.90)	0.0055*	1.30 (0.98–1.72)	0.0686	1.26 (0.95–1.66)	0.1090	1.26 (0.95–1.66)	0.1031
IHD	0.88 (0.68–1.13)	0.3151	0.74 (0.56–0.97)	0.0274*	0.72 (0.55–0.95)	0.0173*	0.72 (0.55–0.95)	0.0187*
COPD	1.90 (1.34–2.62)	0.0005*	1.59 (1.10–2.25)	0.0137*	1.66 (1.15–2.32)	0.0071*	1.77 (1.24–2.48)	0.0023*
ESRD	2.00 (1.17–3.17)	0.0129*	1.91 (1.11–3.10)	0.0220*	2.14 (1.24–3.49)	0.0081*	2.05 (1.19–3.32)	0.0117*
NHYA Fc	1.52 (1.17–1.97)	0.0014*	1.40 (1.08–1.83)	0.0123*	1.49 (1.15–1.95)	0.0029*	1.49 (1.15–1.95)	0.0028*
Baseline LVEF	0.97 (0.83–1.14)	0.7258	1.00 (0.85–1.17)	0.9961				
Post-Treatment LVEF	0.74 (0.63–0.87)	0.0003*			0.67 (0.57–0.79)	<0.0001*		
Change of LVEF	0.68 (0.56–0.84)	0.0003*					0.61 (0.49–0.74)	<0.0001*

*Denotes *P* < .05.

CI, confidence interval; COPD, chronic obstructive pulmonary disease; ESRD, end-stage renal disease; DM, diabetes mellitus; HR, hazard ratio; HT, hypertension; IHD, ischemic heart disease; LVEF, left ventricular ejection fraction; NYHA Fc, New York Heart Association functional class.

### Protocols 2 and 3: Outcomes and Predictors of LVEF Change 6 Months Posttreatment

Of the study patients, improved LVEF was noted in 172 (40.1%), with LVEF increasing from 26.0% ± 6.5% to 47.6% ± 12.2%. Table 5 presents the baseline characteristics of the patients stratified into improved, unchanged, or reduced LVEF groups. The patients with improved LVEF were more likely to have NYHA class III/IV (*P* = .03), higher systolic or diastolic blood pressure (*P* = .05/.02), QRS > 120 ms (*P* = .04), and a smaller LV end diastolic diameter (LVEDD) (*P* < .001). The improved LVEF group had a markedly lower all-cause mortality rate than the other 2 groups did (13.4% vs 23.9% and 29.4%, respectively); and a lower HF readmission rate (48.3% vs 57.7% and 85.3%, respectively). According to the Kaplan–Meier survival analyses, the difference among the improved, unchanged, and reduced LVEF groups (*P* = .02 for all-cause mortality and *P* < .001 for HF readmission) (Figs 2C and 3C) was significant; the patients with improved LVEF had a superior prognosis compared with those with unchanged or reduced LVEF). According to a multivariate Cox proportional hazards model, after adjustment for age, sex, HF, DM, IHD, COPD, ESRD, and NYHA Fc III/IV, posttreatment LVEF remained a statistically significant predictor of future events (HR = 0.59, *P* = .003 for all-cause mortality; HR = 0.61, *P* < .001 for HF readmission). After a multivariate logistic regression with the same adjustments, only LVEDD was an independent predictor of LVEF improvement after 6 months (Odds ratio [OR] = 0.94, *P* < .001) (Table 6).

**Table 5:** Baseline characteristics stratified into improved, unchanged, and reduced LVEF groups.

Characteristics	Improved (n = 172)	Unchanged (*n* = 222)	Worsened (n = 34)	*P*
Gender (male)	116 (67.4)	160 (72.1)	28 (82.4)	0.191
Age (year)	64±15	64±15	65±12	0.731
NYHA class III/IV	106 (61.6)	108 (48.6)	16 (47.1)	0.027*
Comorbidity				
HT	93 (54.1)	111 (50.0))	20 (58.8)	0.531
DM	52 (30.2)	66 (29.7)	14 (41.2)	0.394
IHD	72 (41.9)	92 (41.4)	17 (50.0)	0.636
Previous stroke	19 (38.8)	24 (10.8)	6 (7.6)	0.495
COPD	24 (14.0)	28 (12.6)	3 (8.8)	0.708
ESRD	11 (6.4)	9 (4.1)	2 (5.9)	0.568
Liver cirrhosis	5 (2.9)	9 (4.1)	1 (2.9)	0.814
Biochemistry				
Cr (mg/dL)	1.61±1.6	1.71±2.0	1.62±1.1	0.832
Sodium (mEq/L)	139±3.7	140±3.6	138±4.4	0.121
Vital signs				
SBP (mmHg)	129±23	125±21	120±22	0.046*
DBP (mmHg)	76±17	73±15	69±13	0.016*
Heart rate (bpm)	89±23	84±16	87±18	0.291
Echo findings				
LA diameter (mm)	44.4±8.8	45.2±8.6	47.3±6.3	0.172
LVEDD (mm)	58.3±8.4	63.0±8.6	63.3±7.4	0.000*
Baseline LVEF %	26.0+6.5	26.9±5.9	30.8±3.7	
Second LVEF %	47.6±12.2	28.1±7.1	17.4±3.2	
ECG characteristics				
AF	30 (17.4)	23 (10.4)	5 (14.7)	0.123
QRS duration >120 ms	44 (27.8)	80 (38.6)	14 (46.7)	0.038*
Medication				
Aspirin	74 (43.0)	99 (44.6)	9 (26.5)	0.136
Clopidogrel	34 (19.8)	25 (11.3)	7 (20.6)	0.047*
ACEI or ARB	131 (76.2)	167 (75.2)	21 (61.8)	0.200
Beta blocker	121 (70.3)	149 (67.1)	28 (82.4)	0.191
Digoxin	45 (26.2)	58 (26.1)	13 (12.4)	0.053
Diuretics	128 (74.4)	162 (73.0)	24 (70.6)	0.883
Amiodarone	22 (12.8)	24 (10.8)	2 (5.9)	0.488
Aldosterone antagonist	43 (25.0)	62 (27.9)	10 (29.4)	0.762
All-cause mortality	23 (13.4)	53 (23.9)	10 (29.4)	0.025*
HF readmission	83 (48.3)	128 (57.7)	29 (85.3)	0.000*

*Denotes significance at the *P* < .05 level.

ACEI, angiotensin converting enzyme inhibitor; AF, atrial fibrillation; ARB, angiotensin II receptor blocker; COPD, chronic obstructive pulmonary disease; CRT-D, cardiac resynchronization therapy-defibrillator; DBP, diastolic blood pressure; DM, diabetes mellitus; ESRD, end-stage renal disease; HT, hypertension; ICD, implantable cardioverter defibrillator; IHD, ischemic heart disease; LBBB, left bundle-branch block; LA, left atrial; LV, left ventricular; LVEDD, left ventricular end-diastolic dimension; LVEF, left ventricular ejection fraction; NYHA Fc, New York Heart Association functional class; PPM, permanent pacemaker; SBP, systolic blood pressure.

**Table 6:** Predictors of improved LVEF after standard HF treatment according to logistic regression analysis.

Variable	Univariate OR (95% CI)	*P*	Multivariate OR (95% CI)	*P*
Age, per year	1.00 (0.99–1.01)	0.860		
NYHA Fc III/IV	1.71 (1.15–2.53)	0.007*	1.35 (0.87–2.10)	0.188
IHD	0.97 (0.66–1.44)	0.883		
AF	1.72 (0.99–3.00)	0.056	1.35 (0.74–2.46)	0.334
QRS>120ms	0.59 (0.38–0.91)	0.016*	0.78 (0.49–1.24)	0.287
LVEDD, per mm	0.93 (0.91–0.96)	0.000*	0.94 (0.91–0.96)	0.000*
SBP, per mmhg	1.01 (1.00–1.02)	0.038*		
DBP, per mmhg	1.02 (1.00–1.03)	0.012*	1.01 (0.99–1.01)	0.748
HR, per bpm	1.01 (1.00–1.02)	0.019*		
Beta blocker	1.06 (0.70–1.61)	0.790		
ACEi or ARB	1.16 (0.74–1.81)	0.526		
Aldosterone antagonist	0.85 (0.55–1.32)	0.475		

*Denotes significance at the *P* < .05 level.

**Fig 4:**
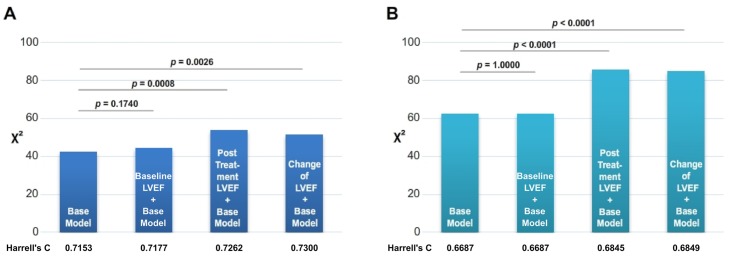
Nested regression models for all-cause mortality (A) and HF readmission (B). The incremental values of baseline LVEF, posttreatment LVEF, and LVEF change were assessed using 3 modeling steps. The first step consisted of fitting a multivariate base model of age, sex, HT, DM, IHD, COPD, ESRD, and NYHA Fc. Baseline LVEF, posttreatment LVEF, or LVEF change was included in the second step. The change in overall log likelihood ratio χ^2^ was used to assess the increase in predictive power after adding LVEF to base model.

The nested regression model results and Harrell’s C statistic indicated that adding posttreatment LVEF to base model yielded a slight increase in predictive power for all-cause mortality (*P* < .001) but that adding baseline LVEF did not (Fig 4A). Similarly, for predicting HF readmission, adding posttreatment LVEF resulted in an incremental increase in predictive power over the base model (*P* < .001), but adding baseline LVEF did not (Fig 4B).

## Discussion

To the best of our knowledge, this is the first report to directly compare the predictive values of baseline LVEF, posttreatment LVEF, and LVEF change for the long-term prognosis of patients with ADHF. This study yielded 3 major results. (1) Posttreatment LVEF was predictive for all-mortality and HF readmission, even after adjustment for age, sex, NYHA Fc, and comorbidities, but baseline LVEF was not. (2) The patients with improved LVEF >10% had the most desirable prognosis, and LVEF change >10% was predictive for all-mortality and HF readmission, even after adjustment for age, sex, NYHA Fc, and comorbidities. (3) Smaller LVEDD was associated with a higher probability of LVEF improvement 6 months after guideline-based HF therapy.

### Baseline and Posttreatment LVEF

Despite continual advances in HF therapeutics, declines in clinical condition necessitating hospitalization remain frequent. Common factors associated with deteriorating HF status include noncompliance with salt and fluid restriction directions, inappropriate drug therapy, infection, ischemia, and arrhythmias [19]. Critically ill patients with ADHF are often admitted to hospitals for emergency treatment, at which time baseline echocardiographic LVEF is typically assessed. However, the effects of medical history as well as lab and examination results obtained at HF patient admission are unclear because the clinical status of patients with ADHF fluctuates widely from the optimal condition. For instance, effective pharmacologic therapies block the activation of the renin-angiotensin system and adrenergic nervous system as well as halt or even reverse the progression of HF to improve clinical outcomes, whereas these improvements may not be present in nonadherent patients with HF. Our study showed that with optimal evidence-based HF therapy, LVEF could improve by up to 70% from baseline enrollment criteria of ≤35%. The difference indicated that using LVEF assessment irrespective of underlying condition may yield widely varying prognostic power; thus, we suggest that clinicians exercise caution in using baseline LVEF for guiding clinical decisions in managing these patients.

### Reversible Dysfunction and Remodeling

In clinical practice, reverse remodeling describes the concept of recovery of myocardial dysfunction after mechanically unloading and restoring neurohormonal overactivation. Reverse remodeling, which is characterized by improved LVEF and reduced LV volume, may occur following myocardial revascularization, timely valve surgery, device therapy such as cardiac resynchronized therapy and ventricular assistance device therapy, and evidence-based medical treatment; it also occasionally occurs spontaneously. Depending on study population and HF etiology, 30%–70% of patients who receive optimal HF therapy exhibit improved LVEF. Crucially, reverse remodeling is closely correlated with long-term benefits in the morbidity and mortality of patients with HF [14,20–23]. Therefore, myocardial remodeling, reflected by changes in LVEF and LV volumes in serial echocardiographic assessments, should be considered a practical prognosis indicator for risk stratification and should guide clinicians’ management plans. For high-risk patients with pathologic remodeling after a reasonable observation period, intensive HF treatment including cardiac surgery or device implantation should be implemented.

In our study cohort of unselective heterogeneous patients with ADHF, approximately 40% of patients had a more than 10% improvement in LVEF 6 months posttreatment and demonstrated superior long-term prognosis in all-cause mortality and HF readmission compared with the groups with unchanged LVEF and a more than 10% decline in LVEF. Analysis using a cutoff value of 10% change of LVEF revealed a significant difference in prognosis between these groups, as shown in a Kaplan–Meier survival curve (*P* = .02). This suggested that some patients could have viable but dysfunctional myocardium. The aforementioned results are well supported by the results of previous clinical trials^11,16^ in which contrast-enhanced cardiac magnetic resonance was used to demonstrate that viable myocardium further predicted LV reverse remodeling. Therefore, LVEF assessed at least 6 months after the initiation of standard HF treatment was a more reliable prognosis predictor than baseline LVEF, which was poorly correlated with long-term outcomes.

LVEF improvement of more than 10% had greater predictive power than did posttreatment LVEF. Several observation studies on the predictive value of LVEF improvement in clinical practice reported different results. Wilcox et al [15] reported that the 4 clinical factors of female sex, no prior MI, nonischemic HF etiology, and no baseline digoxin use are associated with >10% LVEF improvement in unselective patients with HF. Binkley et al [12] suggested that shorter QRS duration, female sex, nonischemic etiology of HF, absence of diabetes, and higher systolic blood pressure are associated with LVEF improvement in patients with dilated cardiomyopathy. Our results are consistent with reports from McNamara et al [13] and Matsumura et al [24], and smaller LVEDD measured at presentation was the strongest predictor of LVEF recovery 6 months posttreatment.

### Effect of Comorbidities on Mortality and HF Readmission

In addition to finding posttreatment LVEF and LVEF change to be crucial outcome predictors, this study revealed the comorbidities of advanced age, HT, DM, COPD, and ESRD to be associated with a higher risk of HF readmission, and advanced age, COPD, and ESRD to be associated with increased risk of all-cause mortality among hospitalized patients with HF. HF is increasingly prevalent among fragile elderly people, and more than one third of patients with HF have noncardiac comorbidities including stroke, DM, chronic kidney disease, and COPD in current HF registries [4]. These conditions contribute to the progression of HF pathologic remodeling through ongoing atherosclerosis processes with myocardial ischemia, refractory fluid overloading and neurohormonal activation, recurrent pulmonary infection, and episodic hypoxic events. It is associated with hospital readmission and overall mortality in patients with HF. In a population-based observation study [25], investigators found that advanced age, male sex, and the presence of comorbidities were associated with poorer survival among patients with HF. O’Connor and colleagues [26] performed posthoc analysis of an HF clinical trial and revealed that approximately half of all deaths and rehospitalization incidents within 60 days of HF hospitalization were secondary to comorbidities rather than deteriorating HF condition. Currently, in-hospital HF care mainly focuses on fluid status management and symptom relief. To reduce the readmission rate and improve survival, broader HF treatment strategies for the prevention and treatment of these comorbidities are required.

### Clinical Implications

Our results suggest that 40% of the patients experienced a significant improvement in LVEF with optimal therapy. LVEF assessed during admission with ADHF was not predictive of outcomes. However, posttreatment LVEF was a reliable prognostic tool and should guide the clinical management for these patients with HF. The strong association between LVEF change and clinical outcomes supports the incorporation of LVEF and LV measures after optimal medical treatment to facilitate the early identification of patients who have not responded to the treatment and who require further aggressive intervention to halt the progression of HF.

### Limitations

Our study had some limitations. First, the data were confined to patients who survived more than 6 months after treatment and who received repeated LVEF assessments, which may limit the scope of our results. However, HF patient death in these 6 months may have mainly resulted from an inevitable clinical course, delayed diagnosis, or inadequate, inappropriate, or untimely treatment; thus, we believe that omitting these patients improved the validity of the data. This investigation focused on the predictability of LVEF after adequate HF therapy. Second, the study cohort was nonhomogenous, with variability in HF etiology, disease duration, and therapy. As with most observation studies, numerous possible uncontrolled confounding factors existed in this study; however, the results still provide new insights on the real-world clinical treatment of patients with HF. Further well-designed randomized studies are required to confirm the results of the present study. Finally, only severe systolic HF patients with LVEF ≤ 35% were enrolled, and whether the current findings may be generalized to patients with mildly impaired systolic function (LVEF 35%–50%) remains to be clarified.

## Conclusions

In this single-center cohort study, we found that posttreatment LVEF but not baseline LVEF was a powerful predictor of both all-cause mortality and HF readmission in patients with ADHF with severely depressed LVEF (≤35%). Improved LVEF (>10% increase) 6 months posttreatment was associated with superior long-term prognosis, and baseline LVEDD was found to be a clinical marker predicting outcome. However, baseline LVEF did not predict cardiovascular outcomes and should not be used as a prognostic factor in clinical practice for patients with ADHF.

## Abbreviations

ADHF: acute decompensated heart failure
COPD: chronic obstructive pulmonary disease
DM: diabetes mellitus
ESRD: end-stage renal disease
HF: heart failure
HR: hazard ratio
HT: hypertension
IHD: ischemic heart disease
LV: left ventricular
LVEDD: left ventricular end-diastolic dimension
LVEF: left ventricular ejection fraction
NYHA Fc: New York Heart Association functional class
